# Galectin-3 as a Novel Multifaceted and Not Only Cardiovascular Biomarker in Patients with Psoriasis with Regard to Systemic Treatment—Preliminary Data

**DOI:** 10.3390/biology11010088

**Published:** 2022-01-07

**Authors:** Anna Baran, Paulina Kiluk, Julia Nowowiejska, Tomasz W. Kaminski, Magdalena Maciaszek, Iwona Flisiak

**Affiliations:** 1 Department of Dermatology and Venereology, Medical University of Bialystok, Zurawia 14 St., 15-540 Bialystok, Poland; paulina.kiluk@umb.edu.pl (P.K.); julia.nowowiejska@umb.edu.pl (J.N.); iwona.flisiak@umb.edu.pl (I.F.); 2 Pittsburgh Heart, Lung and Blood Vascular Medicine Institute, University of Pittsburgh, Pittsburgh, PA 15260, USA; kamins1@pitt.edu; 3 Department of Infectious Diseases and Hepatology, Medical University of Bialystok, Zurawia 14 St., 15-540 Bialystok, Poland; mm.maciaszek@wp.pl

**Keywords:** psoriasis, lectins, galectin-3, comorbidities, cardiovascular biomarker, systemic therapy

## Abstract

**Simple Summary:**

Galectin-3 (gal-3) regulates many different biological processes and diseases, which are common accompanying diseases of psoriasis. Psoriasis is one of the most common skin diseases. There is little information about potential diagnostic role of gal-3 in psoriasis. Serum gal-3 concentrations were measured before and after twelve weeks of antipsoriatic treatment in patients with psoriasis and compared to 11 persons without psoriasis (control group). Serum gal-3 level in patients with psoriasis was significantly higher compared to the control group. In obese patients and long-lasting psoriasis positive relations of gal-3 and index of psoriasis severity were noted. In psoriatics with low gal-3 levels, it was noted that the higher the gal-3, the higher the BMI and glucose level. In patients with long history of psoriasis it was observed that the higher gal-3, the lower the lipids levels. The Gal-3 level might be a factor affecting the course of psoriasis and useful in prediction of cardiometabolic comorbidities, especially in patients with a long history of the disease or obesity. Patients with low serum gal-3 and a short history of psoriasis may have greater risk of diabetes. In obese patients with long-lasting psoriasis, gal-3 may have a beneficial influence against abnormal lipid profiles or perhaps further cardiovascular disorder development.

**Abstract:**

Galectin-3 (gal-3) is a multifunctional regulator of various biological processes and diseases, which are common comorbidities in psoriasis. Data regarding potential diagnostic role of gal-3 in psoriasis are insufficient. Serum gal-3 levels were evaluated before and after twelve weeks of treatment with acitretin or methotrexate in 31 patients with plaque-type psoriasis and compared to 11 healthy control group. The mean serum galectin-3 level in patients with psoriasis was significantly higher compared to the control group (*p* < 0.01). In patients with obesity and long-lasting psoriasis (>20 years) positive relations of gal-3 and PASI were noted. In psoriatics with low gal-3 levels, positive correlations between the gal-3 and BMI, glucose level, and with the latter in short-lasting psoriasis (<20 years) were noted. In the long history of psoriasis, gal-3 was negatively correlated with lipids levels. The Gal-3 level might be a multifaceted modulator of the course of psoriasis and predictive factor of cardiometabolic comorbidities’ development, especially in patients with a long history of the disease or obesity. Patients with low serum gal-3 and short history of psoriasis are presumably at greater risk of diabetes. In patients with long-lasting psoriasis and concomitant obesity, gal-3 may exert a protective role against dyslipidemia or perhaps further CMD development.

## 1. Introduction

Psoriasis is a common chronic, autoimmune, and inflammatory disease affecting 2–4% of the global population and an incidence of 60.4 to 140 new cases per 100,000 [1,2]. In recent years the viewpoint on the pathogenesis of psoriasis has evolved significantly at the same time due to continuous enriched research in this field. Thus, currently psoriasis is definitely more than skin deep and closely related to numerous cardiometabolic disorders (CMDs), including obesity, diabetes mellitus (DM), dyslipidemia, and hypertension, which make up the metabolic syndrome (MS), as well as non-alcoholic fatty liver disease (NAFLD) and cardiovascular diseases (CVD) [2,3,4]. It has been widely proven that the higher rate of comorbidities is related to psoriasis severity [5,6]. Persons with psoriasis have shortened life expectancy of over 5 years mainly due to CVD and increased relative risk of mortality of 1.53 in comparison to 1.12 in the general population [7]. Psoriatics with severe form present a higher rate of ischemic heart diseases but also have additional absolute risk of 6.2% of a major adverse cardiovascular event (MACE) within 10 years of psoriasis history compared to the healthy population [2,8]. The multidirectional relationship of psoriasis with numerous comorbidities is translated by common genetic or immunological pathways, oxidative stress, as well as systemic metabolically driven inflammation which is important in psoriasis pathogenesis and consequently leads to the development of atherosclerosis, insulin resistance, and CMDs [3,4,5]. For years, researchers have been constantly looking for novel modulators of metaflammation in psoriasis to reduce the risk of internal disorders or use as potential targets for therapeutic intervention. We also previously investigated various active proteins in serum of psoriatic patients and demonstrated them as novel indicators of inflammation or the metabolic complications development in psoriasis [9,10].

According to recent accumulating evidence, galectins seem to be very promising molecular effectors mediating a wide range of biological processes in various tissues including cutaneous and involved in pathogenesis of various diseases [11,12]. Galectins are a growing family of β-galactoside-binding lectins consisting of either one or two 130 amino-acid-long highly conserved carbohydrate recognition domains (CRD). At least 15 galectins have been described in mammalians so far and they have been divided into three groups based on their CRD number, structure, and properties [11,13,14,15]. Galectins, by binding saccharides in glycoconjugates on the cell surface, exert multimodal biological roles, not yet fully known, in the regulation of cell–cell and cell–matrix interactions, immunity, expression of certain genes, regeneration, apoptosis, and cancer progression [11,12,14,16,17]. It is noteworthy that the molecules can act opposite, depending on various conditions, serving as positive or negative modulators of the same processes [12,13,14]. The plasticity of galectins and their wide distribution in tissues (not only in the cytoplasm and nuclei but also in the extracellular space) makes them extremely interesting for research but also clinical usability. Many galectins take part in cancers’ invasiveness, and have been related with higher rates of metastases and poorer survival outcome in different cancers [11,18]. Galectin-7 has been linked not only with lung cancer, but also with being highly expressed in epithelial tissues, and related to the regulation of skin homeostasis or keratinocytes proliferation [18,19]. Chen et al. demonstrated that galectin-7 mRNA was downregulated in human psoriatic lesions and a IL-23-induced psoriasis model that proves the role of the lectin in pathogenesis of psoriasis [19].

Galectin-3 (gal-3) is the only representative of chimera-type galectin, of a 35-kDa weight, encoded by the LGALS3 gene located on chromosome 14 [13,14,20]. It has a wide tissue distribution with high expression in epithelial and myeloid cells, but also in these involved in immune response, such as neutrophils, eosinophils, monocytes, mast cells, Langerhans cells, dendritic cells, osteoclasts, chondrocytes or activated macrophages, and T- and B-cells [13,15,16]. With reference to the skin, its expression is high in keratinocytes, hair follicles, and sebaceous and sweat glands, but also secreted by other resident skin cells such as melanocytes, fibroblasts, and monocytes [15,17]. Depending on the localization of galectin-3—extra- or intracellular—it displays different functions [13]. Gal-3, even most other galectins, is involved in cell adhesion to extracellular matrix, modulation of cell migration and adhesion, and inhibition of apoptosis. It is a significant regulator of signal transduction and immune surveillance, cell growth, and differentiation, including epidermal keratinocytes. Further, gal-3 promotes angiogenesis and induction of fibrosis [16]. Similarly to other lectins, gal-3 is a pro-tumor factor enhancing oncogenesis and metastasis [13]. Its higher expression was noted in various tumors such as of the thyroid gland, the central nervous system, the kidney, the liver, and lymphoma or melanoma [13]. The multifunctional activity of gal-3, as well as in the skin, results in great interest over other galectins for its role in various clinical conditions and diseases, such as asthma, cancer, atherosclerosis, CVD, obesity, diabetes mellitus, kidney disorders, atopic dermatitis, and psoriasis, as well. Much research has demonstrated rapid expression of gal-3 in biological fluids from injured or inflammatory cells under diseased conditions indicating the lectin as a sensitive regulator of many critical and pathological processes and an active promotor of inflammation [14,20,21]. Furthermore, gal-3 has been identified as a next-generation marker for detection of early stages of different diseases inter alia myocardial dysfunction and heart failure (HF) or cardiac degeneration in acute myocarditis [14,22,23]. Numerous data proved that elevated serum galectin-3 is related with recurrent HF, poorer prognosis of coronary heart disease and increased risk of death [13,20,23,24]. In 2014, the Food and Drug Administration (FDA) identified gal-3 as one of the validated cardiovascular biomarkers [21]. Further, lectin is strongly linked with atherosclerosis which also is closely related to psoriasis [20,21]. An increased gal-3 level was positively correlated with carotid intima media thickness which is again also greater in psoriasis especially with concomitant metabolic syndrome [25,26]. Therefore, the gal-3 assessment might be extremely useful in diagnosis and prediction of CMDs in psoriasis. Data on this subject are scarce so far. Kotwica et al. suggested that enhanced profibrotic activity reflected by elevated serum gal-3 may be involved in subclinical myocardial impairment in psoriasis [27]. Lacina et al. investigated the expression of several galectins in the psoriatic epithelium which did not express gal-3 contrary to normal epidermis [28]. Interestingly, strong expression of galectin-3/galectin-3-reactive glycoligands in capillaries of psoriatic plaques pointed that altered galectin expression and binding patterns in psoriatic skin indicates its modifying impact on keratinocyte maturation in hyperactivated psoriatic epithelium and thus on the development of psoriatic skin lesions [28].

The wide range of multifunctionality of gal-3 inter alia being a biomarker of inflammation, atherosclerosis, fibrosis, oxidative stress, and many others, involved intensively in the pathogenesis of psoriasis, has prompted us to investigate the potential diagnostic and clinical usefulness of gal-3 in serum of psoriatics and its relationship with the disease severity, metabolic, or inflammatory indicators. We aimed to look for various interrelations between gal-3 levels and various parameters in terms of duration of psoriasis to clarify whether the lectin may be a factor stratifying the risk of developing complications. Furthermore, to the best of our knowledge, we are one of the first who evaluated the impact of classic systemic antipsoriatic treatment on gal-3 levels in order to assess its potency to assess the efficacy of certain drugs in psoriasis or perhaps contribute to develop newer therapeutic strategies.

## 2. Materials and Methods

Our prospective study included 31 adult patients (19 males and 12 females) with flares of plaque-type psoriasis, and 11 sex-, age-, and BMI-matched volunteers without dermatoses. Patients with other types of psoriasis, pregnant, breast-feeding, chronic metabolic or inflammatory diseases, and current or 5 years’ history of any neoplasms were excluded from the study. None of the patients was under dietary restrictions or received any antipsoriatic treatment for 1 month before the inclusion to the study. The psoriasis area and severity index (PASI) was calculated by the same dermatologist in all patients. The study group was divided according to PASI into 3 subgroups: mild (PASI 1), meaning scored under 10 points noted in 8 patients, moderate (PASI 2)—PASI 10–20 points evaluated in 12 subjects, and severe psoriasis (PASI 3) was related to PASI > 20 points calculated in 11 persons. Body mass index (BMI) was evaluated as weight/height ^2^ (kg/m^2^). The study group was further divided according to BMI: Group 0 meant the control group; BMI 1—10 normal weight (18.5–24.9) patients with psoriasis, BMI 2—indicated 10 overweight psoriatics (BMI 25–29.9), BMI 3—obesity (BMI > 30), noted in 11 patients. Levels of C-reactive protein (CRP), complete blood count (CBC), serum glucose, total cholesterol (Total Chol), HDL (high-density lipoprotein), LDL (low-density lipoprotein), triglycerides (TG), and indicators of kidney and liver functions were evaluated in the study and control group before and after therapy in psoriatics. Blood samples were collected before and repeated after twelve weeks of systemic treatment with 15 mg/week of methotrexate (MTX) (18 patients) or acitretin (ACY) (13 persons) in a bodyweight dose of 0.5 mg/kg/day, considering patients’ internal condition, tolerability, and indications. All participants gave their signed informed consent before the initiation. The study was approved by the local bioethical committee (protocol number R-I-002/354/2015) and was performed in accordance with the principles of the Helsinki Declaration.

### 2.1. Serum Collection

Fasting blood samples were taken from the study and control group, using vacutainer tubes and allowed to clot for 30 min. Samples were centrifugated for 15 min at 2000× *g*, and then separated serum was frozen immediately and preserved at −80 °C until analysis. Blood samples for biochemical tests and blood counts were collected at the same time to other tubes and were performed by routine laboratory techniques using an automated analyzer. Serum level of assay parameters were measured using validated and calibrated Bio-Plex 200 System provided by Bio-Rad. For the measurement of certain magnetic bead-based assays among other galectin-3, Bio-Plex Pro RBM Human Metabolic Panel 2 was used.

### 2.2. Statistical Analysis

The normality of distribution of the obtained data was tested using the Shapiro–Wilk test and quantitative data were expressed as mean ± SEM. The non-Gaussian values were shown as a median with the full range. The Student’s *t*-test or nonparametric Mann–Whitney test were used to compare differences between analyzed two distinct groups, whereas for binary data, the Chi-square test was used. The analysis of variance (ANOVA) or Kruskal–Wallis test was used to evaluate differences between the study subgroups and followed by Bonferroni post hoc analysis when appropriate. The correlations between the variables were calculated using Spearman’s rank correlation analysis. A two-tailed *p*-value < 0.05 was considered to be statistically significant. Computations were performed using GraphPad 9 Prism Software and the power of the analysis and group sampling were estimated using StatMate 2 Software (both GraphPad Software Inc., La Jolla, San Diego, CA, USA).

## 3. Results

The study included 31 patients with a flare of plaque-type psoriasis, 12 women and 19 men, with the mean age of 51.4 ± 9.61, and 11 healthy volunteers of the mean age of 55.2 ± 15.6, matched for age, weight, and BMI. The mean value of BMI of the patients was 28.5 ± 6.53 kg/m^2^. Severity of psoriatic skin lesions expressed by mean PASI score was 17.15 ± 7.92 before treatment and 4.31 ± 2.06 after therapy. Baseline characteristic of the control group and patients are summarized in Table 1 and Table 2. Additional, more accurate analysis of the psoriatic subjects subdivided with regard to the specific drug they were treated with, along with division according to BMI and PASI, has been placed in the Supplementary Materials.

**Figure 1:**
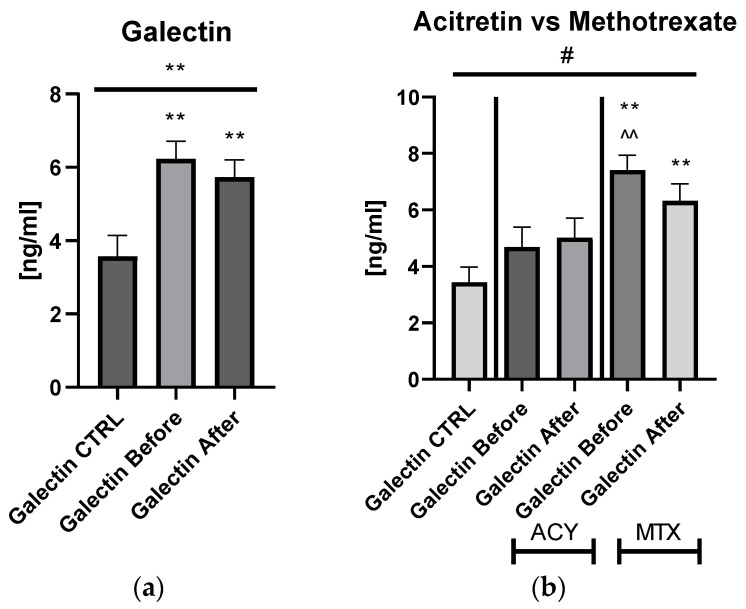
The levels of gal-3 in psoriatics before and after total treatment (**a**) compared to control group and divided into subgroups undergoing therapy separately with acitretin and methotrexate (**b**). **—means the existence of statistically significant difference between patients single group compared to control group with *p* < 0.05; <0.01, respectively. ^^—means the difference between ACY and MTX subgroups before treatment with *p* < 0.01. #—shows the statistical significance between control group and marked patients’ subgroups when compared using ANOVA with *p* < 0.05.

The mean of serum galectin-3 concentration in patients with psoriasis was 6.23 ± 0.487 ng/mL before treatment and after 5.74 ± 0.461 ng/mL, and was significantly higher compared to the control group: 3.44 ng/mL ± 0.537 ng/mL (*p* < 0.01) (Figure 1).

**Figure 2:**
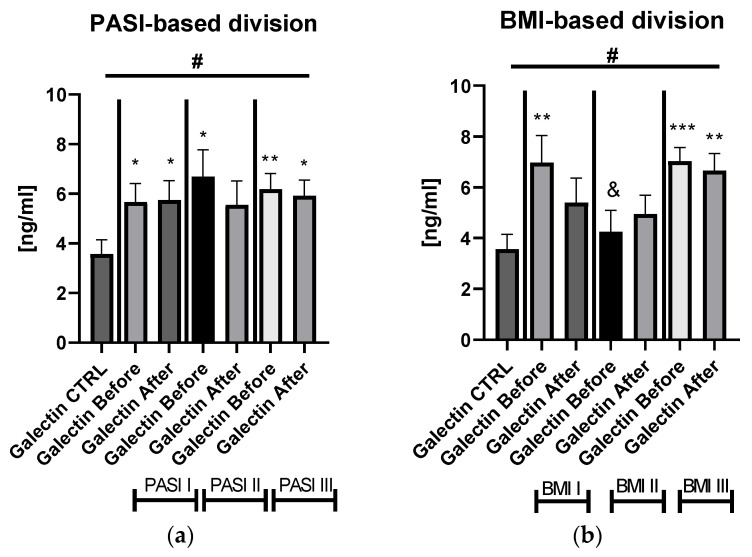
The levels of gal-3 in psoriatics before and after total treatment compared to control group divided into subgroups based on PASI (**a**) and BMI (**b**). */**/***—means the existence of statistically significant difference between patients single group compared to controls with *p* < 0.05; <0.01, <0.001 respectively. &—means the existence of trend (*p* < 0.1) between BMI I and BMI II subgroups before treatment. #—shows the statistical significance between controls and marked patients’ subgroups when compared using ANOVA with *p* < 0.05 and mean trend.

Serum galectin-3 level did not correlate with psoriasis severity expressed with PASI score (*p* > 0.05) (Figure S1). After dividing the study group depending on the PASI score, the lectin concentrations were significantly higher in subjects with all three PASI subgroups before treatment, compared with the control group and remaining on similar level after therapy with statistical significance excluding the mild-to-moderate group (Figure 2a).

Significant differences in gal-3 levels between the control group and all three PASI, BMI, and patients on ACY or MTX subgroups, before and after treatment, were achieved in the ANOVA test (Figure 2a,b). Gal-3 levels did not correlate with total BMI in psoriatics before treatment (*p* > 0.05) (Figure S1). Regarding the BMI subgroups, the serum gal-3 level was significantly elevated in all three study subgroups, with the highest level in obese patients (*p* < 0.001) (Figure 2b).

**Figure 3:**
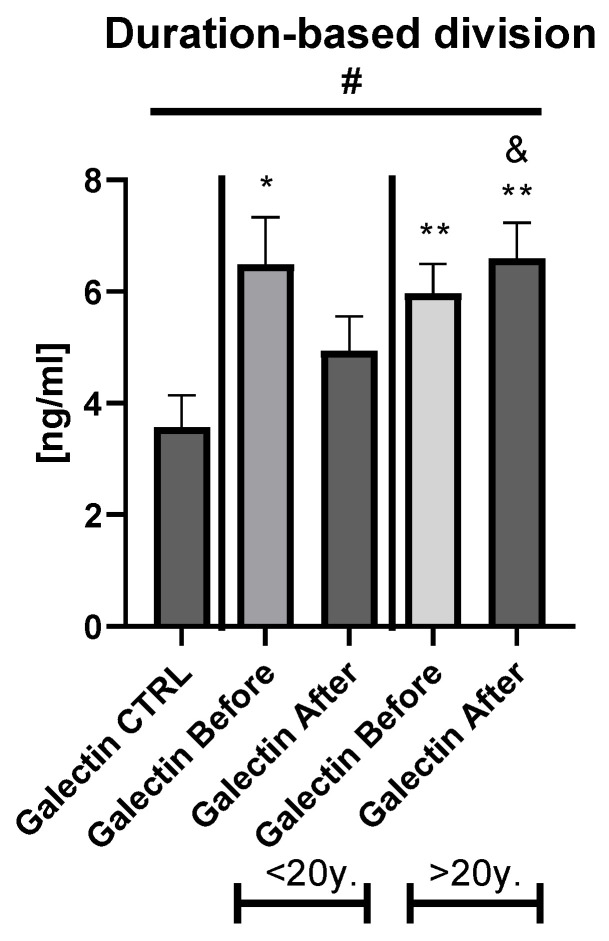
The levels of gal-3 in psoriatics before and after total treatment compared to control group depending on the duration of psoriasis. */**—means the existence of statistically significant difference between patients single group compared to control group with *p* < 0.05; <0.01, respectively. &—means the existence of trend (*p* < 0.1) between <20 y. and >20 y. subgroups after treatment #—shows the statistical significance between control group and marked patients subgroups when compared using ANOVA with *p* < 0.05 and mean trend.

With reference to closer characteristics of the patients divided into subgroups regarding BMI, subjects with obesity were older, smoked significantly more often, had significantly elevated glucose and TG level, and higher ALT activity before and after treatment compared to psoriatics of normal weight or overweight ones (Table S2). With regard to the division of the study group into PASI subgroups, patients with severe psoriasis had significantly higher weights, BMI, and CRP levels before and after therapy in comparison to the control group (Table S3). The duration-based division of the patients’ group with a threshold of 20 years revealed that in both short- and long-lasting psoriasis, the gal-3 level was significantly increased in comparison to the healthy persons (Figure 3).

**Figure 4:**
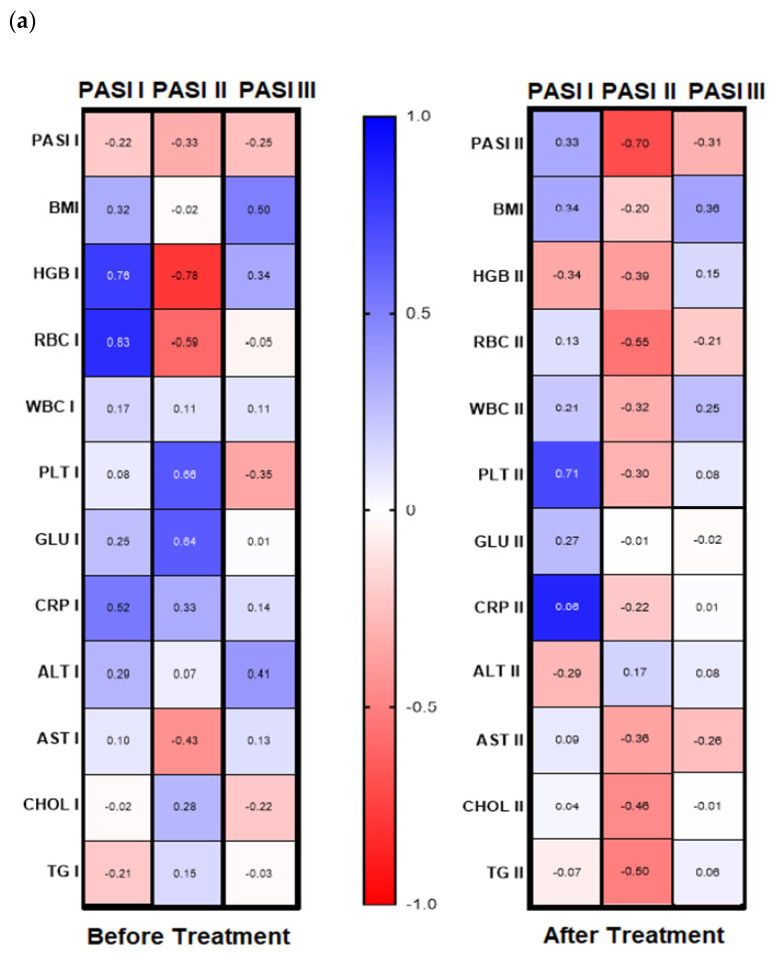

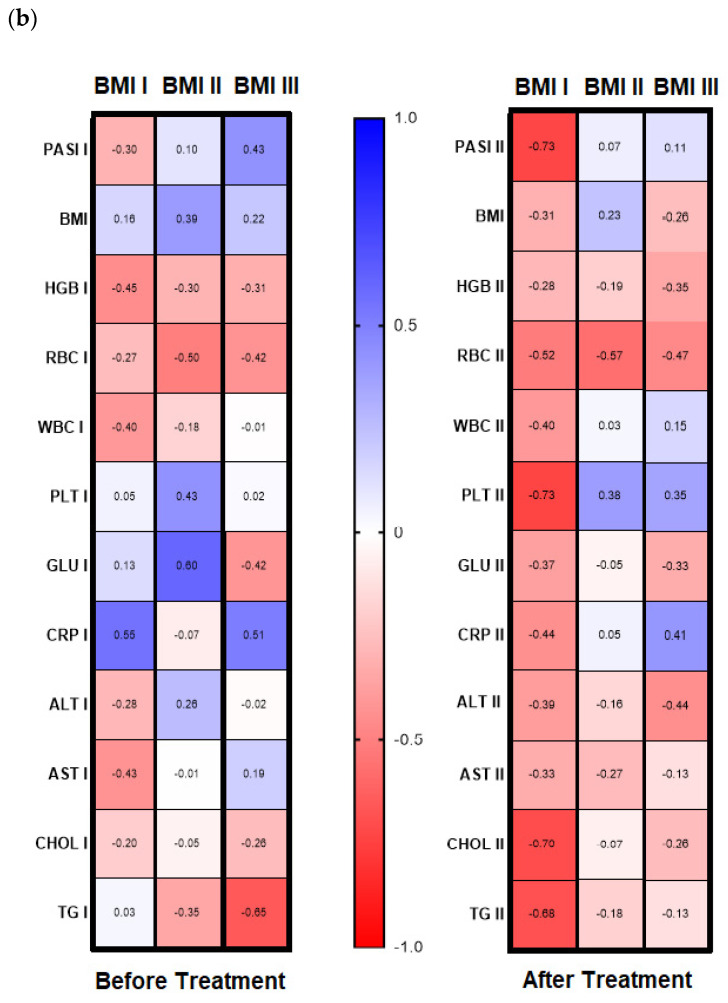
Correlations before and after total treatment between selected parameters and gal-3 level inside PASI (**a**) and BMI (**b**) subgroups with use of Spearman’s rank correlation.

Analyzing possible correlations between studied lectin and demographic, clinical, or laboratory parameters of the patients, including inter alia metabolic or inflammatory indices such as CRP, WBC, glucose level, lipids profile, BMI, no statistical importance was noted before the therapy (all *p* > 0.05) (Figure S1). However, after subdivision of the study group regarding PASI, BMI some correlations were noted (Figure 4a,b). Before therapy, in patients with mild psoriasis (PASI I) gal-3 positively correlated with HGB, RBC, and CRP levels (Figure 4b, left panel). In subjects with mild-to-moderate psoriasis, negative relations between the lectin and HGB, RBC, and AST activity were found while in most diseased patients gal-3 positively correlated with BMI and ALT (Figure 4b, left panel). Regarding selected relations inside the BMI subgroups, we noted that in psoriatics with normal weight (BMI I) before treatment gal-3 positively correlated with CRP, and inversely with levels of HGB, WBC, and AST activity (Figure 4, left panel). In overweight (BMI II) psoriatics, gal-3 levels positively correlated with PLT and glucose level and negatively with RBC (Figure 4b, left panel). In obese patients (BMI III), before treatment a positive relationship of the lectin level and PASI and CRP were noted, when with RBC, GLU, and TG negative correlations were reported (Figure 4b, left panel).

**Figure 5:**
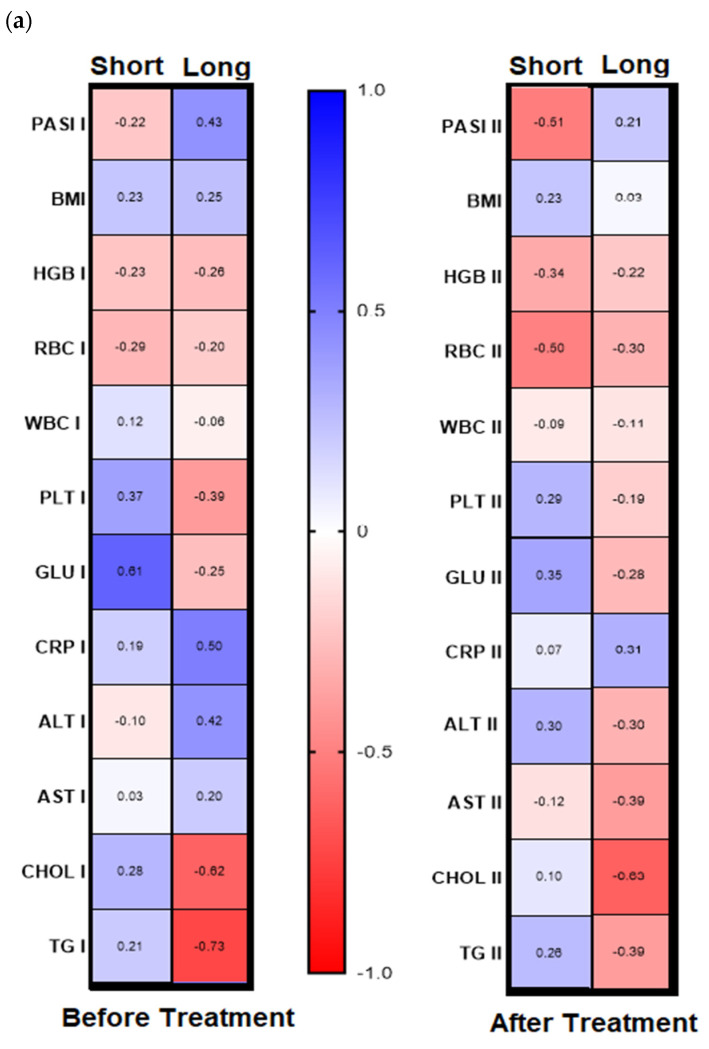

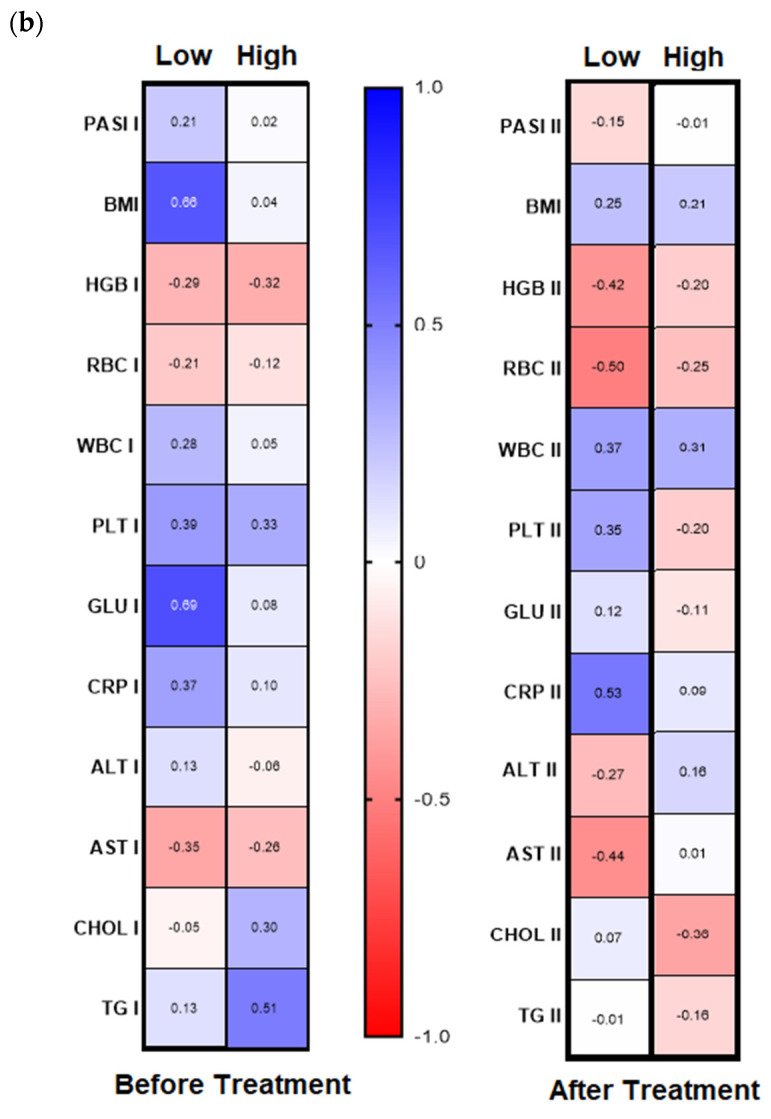
Chosen correlations before and after treatment with reference to duration of psoriasis (**a**) and the galectin-3 concentration threshold (**b**) with use of Spearman’s rank correlation.

Analyzing further dependencies before treatment, the division of the patients group into gal-3 serum level with its threshold of 6 ng/mL was revealed in psoriatics with low lectin level positive significant correlation of gal-3 with BMI and glucose level and with the latter in short-lasting psoriasis (<20 years, *n* = 15) (Figure 5a,b, left panels, Table S6a,b). In turn, in patients with long-lasting psoriasis (>20 years, *n* = 16) negative correlations between gal-3 levels and total cholesterol and triglycerides levels were noted, and positive ones with PASI, CRP, and ALT activity (Figure 5a,b, left panels, Table S6a).

After twelve weeks of systemic therapy, a significant clinical improvement was observed along with decrease in inflammatory indicators levels (Table 1). The mean gal-3 serum level did not change significantly, only slightly decreased, remaining statistically higher than of the control group after two drugs in total (*p* < 0.001) (Figure 1a). Further, after division into subgroups of patients treated with the drugs separately, no meaningful changes in gal-3 levels occurred (both *p* > 0.05) (Figures S1b and S4a,b). Serum gal-3 concentration was constant in subjects on acitretin and stayed of a similar value to that in the controls (*p* > 0.05). Interestingly, in patients on methotrexate, gal-3 was significantly higher than in the control group and in subjects on acitretin before treatment, decreasing insignificantly, and remaining higher than in the control group at statistical significance (all *p* > 0.01) (Figures S1b and S4a,b). There were no important differences in galectin-3 levels between three PASI subgroups after treatment (Figure 2a). According to BMI, the gal-3 level remained significantly higher in obese patients after treatment, while in psoriatics of normal weight it lost its significance and in overweight subjects the levels were similar to those of the controls in the primary and post-treatment (Figure 2b). After therapy in patients with a short-length history, the gal-3 level decreased, losing its statistically higher level comparing to the controls (*p* < 0.05), while in patients with longer-lasting psoriasis the gal-3 level had an upward trend and stayed still significantly elevated in comparison to the control group (*p* < 0.01) (Figure 3).

Analyzing the correlations between gal-3 and selected clinical and laboratory parameters, no significant relations were noted after therapy beside a negative one with RBC level (Figure S3). However, inside the PASI subgroups after treatment, we found a positive relation with PLT and CRP in patients with mild psoriasis (Figure 4a, right panel). In PASI II subgroup after therapy, negative correlations with PASI, RBC, Chol, and TG were noted (Figure 4a, right panel).

With reference to the BMI subgroups, after twelve weeks of systemic treatment, previously observed correlations changed differently. In psoriatics of normal weight, gal-3 level negatively correlated with PASI, PLT, Chol, and TG (Figure 4b, right panel). In all three BMI subgroups gal-3 negatively correlated with RBC after therapy. Additionally, in obese subjects, a negative relation between the lectin and RBC and ALT activity was noted and positive with CRP (Figure 4b, right panel). Further, after therapy in patients with low gal-3 levels, negative correlations between the lectin and HGB, RBC, and AST and positive with CRP were noted (Figure 5b, right panel). In subjects with short-lasting psoriasis, after therapy gal-3 negatively correlated with PASI and RBC while in the case of long-lasting disease, gal-3 was negatively related with Chol and TG levels, similarly as before treatment (Figure 5a, right panel, Table S6a,b).

## 4. Discussion

In the last decades more and more scientific attention has been increasingly drawn to the multifunctional proteins such as galectins, especially gal-3. Deepening knowledge about the increasing diagnostic usability of gal-3 along with newer discovered properties in many different clinical contexts and biological processes, such as growth, proliferation, inflammation, apoptosis, oxidative stress, immunity, metabolism, makes gal-3 unique, and extraordinary marker to be explored. Furthermore, it has not only implications in early detection of various diseases, their pathogenesis or prognosis, but also galectin-modulating drugs are becoming promising.

Gathering the current knowledge and future perspectives of a novel approach to the diagnostics and antipsoriatic therapy prompt us to explore the multimodal potential role of gal-3 in patients with psoriasis. Furthermore, measurement of gal-3 might provide valuable information in CVD prognosis and risk stratification in patients with psoriasis. However, the data in this field are very scarce and ambiguous, and thus our results are extremely difficult to discuss, and the conclusions drawn should be considered preliminary, but at the same time innovative.

Gal-3 has been found in cytoplasm of normal keratinocytes with perinuclear enhancement in the basal and the suprabasal layers [16,29]. Importantly, particularly in terms of participation in the pathogenesis of psoriasis, upregulation of galectin-3 is involved in differentiation and maturation of keratinocytes [15,16].

In the study of Lacina et al., gal-3 expression was lower in the psoriatic epithelium in contrast to normal epidermis [28]. Furthermore, the authors demonstrated a strong expression of galectin-3/galectin-3-reactive glycoligands in capillaries of psoriatic dermis reflecting the activation of endothelium in the disease. The research demonstrated that the altered gal-3 expression and binding pattern in psoriatic skin indicates the modified process of keratinocyte maturation in hyperactivated psoriatic epidermis. Furthermore, the upregulated expression of galectin-3 in dermal capillaries of psoriatic skin can be important for dermal capillary rearrangement and migration of inflammatory cells to psoriatic skin [28]. Shi et al. also reported lower expression of gal-3 in psoriatic lesions, but not in the normal epidermis [30], similar to another paper by Shi et al. [31]. Importantly, the authors observed a great improvement of imiquimod-induced psoriasiform dermatitis in gal-3 −/− mice after restoration of the lectin in the skin by intracutaneous injection of recombinant human gal-3 [30]. These data are among the fundamental evidence of the common relationship between gal and psoriasis. Furthermore, they point to potential novel therapeutic approach in psoriasis.

We found only three research papers evaluating serum gal-3 levels in psoriatic patients which we can compare our results with. However, to the best of our knowledge, there is no single study assessing gal-3 levels in psoriasis in relation to its systemic therapy. We demonstrated that serum galectin-3 concentration was significantly increased in patients with psoriasis comparing to the healthy persons. Given the undeniable fact that gal-3 is a marker of inter alia inflammation, oxidative stress, atherogenesis, angiogenesis, along with our significantly higher level in psoriatics, we can speculate that gal-3 levels might be a multidirectional inductor or stimulator of the mentioned processes involved in the pathogenesis of psoriasis. Significant elevation of serum gal-3 level was also noted in diseases related with skin other than psoriasis, such as Behcet’s disease, limited cutaneous systemic sclerosis, or lupus erythematosus [32,33,34].

Hayran et al. found gal-3 to be elevated in psoriatics compared to the control group, which corresponds with our outcomes [35]. In contrast to our results, Özden et al. demonstrated significantly lower serum galectin-3 level in patients with psoriasis. They did not report any relation of the lectin with psoriasis severity expresses by PASI score even after division according to PASI subgroups [36]. Although our results are contradictory, differences should be noted in the demographic and clinical data of the study groups. The authors of the cited paper had similarly numerical results to our group of psoriatics; however, the mean PASI was much lower than thst of our patients; moreover, the mean age of the study and control groups also varied [36]. Perhaps other factors such as genetic background, diet, or experimental nuances have impact on the serum gal-3 level. Furthermore, gal-3 plasticity may provide at least bimodal action, not yet clear, especially in psoriasis. Galectin may be protective but also have negative consequences. As in the case of diabetes, divergent data have stated gal-3 as a protective molecule and others demonstrated its role in progression to diabetes and vascular complications [13,17,37].

Kotwica et al. investigated the association between gal-3 level—a recognized mediator of fibrosis with inflammatory activation and left ventricular (LV) systolic and diastolic function in patients with psoriasis. They noted, as we did, significantly elevated serum gal-3 levels and suggested that this upregulation together with inflammation modulated the subclinical LV systolic dysfunction in psoriasis [27]. The data cited, as well as our own results and the FDA approval of gal-3 as a cardiovascular biomarker, confirm that galectin-3 is a significant multifunctional biomarker of psoriasis and its comorbidities, especially myocardial. We tried to look for the various relationships dividing the study group regarding BMI, PASI, duration of psoriasis, as well as gal-3 concentration threshold. As gal-3 was related positively with glucose level in short-lasting psoriasis, we hypothesized that lectin might be useful for detecting diabetes in early stages of both diseases. In patients with a long history of gal-3, it loses these properties. As mentioned above, the links of gal-3 with diabetes are still controversial and seem to be bidirectional; thus, further studies are necessary. Referring to the literature, patients with gal-3 level > 25 ng/mL had much higher risk of various complications from nephropathy through myocardial infarction to peripheral artery disease than patients with gal-3 level < 10 ng/mL [38]. In other research, gal-3 was proposed as a marker of stratification for therapy. Patients treated with rosuvastatin with low gal-3 levels (<19 ng/mL) had better survival rates and lower cardiovascular event rates [39]. The results concerning a certain threshold of gal serum level which might be considered as stratification marker in psoriatics patients at low or high risk for cardiac or other internal complications are somehow confusing. However, it may be hypothesized that patients with short-lasting psoriasis and low gal-3 concentration are at higher risk of carbohydrate metabolism disorders development. In patients with long history of psoriasis gal-3 seems to exert protective role against dyslipidemia. However, at the same time promotes inflammation, severity of psoriasis, and perhaps liver dysfunction, especially in most diseased patients. These complicated results point to uncertain and bimodal action of gal-3 but also, to be elucidated, interrelations between the protein and lipid and carbohydrate metabolism. Presumably in long-lasting psoriasis, the lectin through some compensatory mechanism may be a part of an adaptive response to more chronic metaflammation.

In severe psoriatics, gal-3 positively correlated with BMI and in obese ones with the disease severity index and CRP, which further emphasizes the links with adipose tissue metabolism and promoting inflammation in psoriasis. Thus, gal-3 might be an inflammatory marker and of psoriasis severity in obese patients. Furthermore, it is worth noting the multiple correlations of gal-3 with blood morphotic indicators recurring in many of our divisions of study group. They are not obvious and they indicate some links of gal-3 with erythropoiesis in psoriasis, but this requires further research.

Analyzing the impact of the systemic treatment in total and with both drugs separately we did not notice any important influences on serum gal-3 levels which might suggest inter alia its insufficient cardioprotective effect beside achieving significant clinical improvement. Moreover, we proved that gal-3 level cannot be a useful a marker of effectiveness of classic systemic treatment of psoriasis.

Further, in obese patients, after therapy gal-3 was related positively with CRP and negatively with ALT which points to the lectin’s involvement in inflammation promotion and liver activity in relation to adiposity and antipsoriatic treatment. Interestingly, analyzing the associations regarding the duration of the disease after treatment, as the significant negative relation of gal-3 with lipids levels persisted in patients with long-lasting psoriasis, it seems to be a protector together with the treatment against dyslipidemia in those psoriatics.

Interestingly, in patients with short-term disease, gal-3 level decreased after the therapy, losing its statistical difference in comparison to the control group, while in long-lasting psoriasis its further increase was observed. This reflects the better effect of the systemic therapy in patients with shorter psoriasis in terms of inhibiting, however insignificantly, the development of various pathogenic processes and comorbidities via downregulation of gal-3 level. Non-significant results achieved after treatment point to undoubted other modifiers, exacerbators, or modulators which disrupt the unclear interplay between gal-3, CVD and psoriasis with regard to its therapy as well. Additionally, in other words, the results obtained importantly suggest the role of gal-3 in intensifying within the duration of psoriasis chronic inflammation and its consequences which influence the therapy effectiveness. Perhaps administration of other systemic drugs or biologicals would be more significant in gal-3 lowering. Recently, a new therapeutic option with the use of gal-3 inhibitors has been proposed and introduced [16]. Unexpectedly, in a female patient with nonalcoholic steatohepatitis with concomitant psoriasis during phase 2 clinical study on gal-3 inhibitor (GR-MD-02) a complete remission of the skin lesions was noted [40]. Further, Ritchie et al. evaluated the efficacy and safety of the aforementioned gal-3 inhibitor in five psoriatic patients [41]. After 6 months of the therapy, an average 51.9% PASI reduction was achieved. The results are not very optimistic, but the study group was very little with no control group, and further randomized controlled clinical trials and dose-finding research are definitely needed in order to define the galectin-3 inhibitors’ actual and potential use for treating psoriasis. However, looking at many clinical trials, now ongoing with use of different gal-3 antagonists for various clinical conditions, such as chronic inflammation and fibrosis in the lungs or the kidney, osteoarthritis, atopic dermatitis, melanoma, and others, emphasize outstanding multidirectional role of gal-3 level in plenty of biological processes and promising use of its inhibitors in the perspective of future therapies for numerous diseases, including psoriasis [13,16].

The limitations of the presented study result from, among others, small studied groups and more subgroups, contradictory results, and the descriptive clinical nature of the paper. We plan to expand our study group, use other systemic drugs, and perhaps in the future we may be able to participate in a trial on gal-3 inhibitors for potential therapeutic purposes in psoriasis. Gathering the results obtained, we admit that they are very complex, with a difficult to unambiguous assessment, and thus they should be considered as preliminary, but at the same time extremely intriguing and motivating to further investigations.

Concluding, on the one hand, gal-3 has the best-known multidimensionality and multifunctionality in humans. On the other hand, its plasticity and sparse data on psoriasis require in-depth research. Based on scientific evidence along with own outcomes, gal-3 seems a fascinating multifaceted marker of psoriasis and its various pathological processes and comorbidities development and could be considered at least, but not only, as a cardiovascular biomarker in psoriasis. Gal-3 cannot serve as an indicator of psoriasis severity nor treatment effectiveness. However, as it correlated positively with PASI in obese psoriatics, gal-3 may be a marker of the disease intensity in this group.

Further, in long-lasting disease, gal-3 might be a marker of inflammation, psoriasis severity, and perhaps liver dysfunction. Presumably, the lectin could be perceived as a double-edged sword in psoriasis depending on various pathological conditions, concentration thresholds, or the disease duration. Patients with low serum gal-3 concentration (<6 ng/mL) and short history of psoriasis seems to be at greater risk of carbohydrate metabolism disorders development; thus, gal-3 may serve as a potential detector for prediabetes and diabetes, especially in these groups of patients. In patients with long-lasting psoriasis and obese ones, gal-3 may be a compensation or protective marker against dyslipidemia or perhaps further CMDs development. Numerous different relations between gal-3 levels and morphotic blood components highlight the interplay with disturbed erythropoiesis in psoriasis, but further studies are required. Classic systemic therapy seems not to significantly affect gal-3 level in psoriasis; however, methotrexate results in its downregulation which points to more beneficial cardioprotective impact of the latter comparing to acitretin. Furthermore, a stronger effect on lowering gal-3 of systemic therapy in patients with shorter psoriasis, however insignificantly, on the one hand reflects its beneficial impact on the inhibition of various pathogenic processes, but on the other hand, stresses the need for early implementation of systemic treatment of psoriasis to prevent the development of comorbidities. The complex results obtained, still evaluating knowledge about gal-3 along with promising data on its inhibitors, should arise further attempts to extensive research on this extraordinary lectin and presumably develop new gal-3 targeted therapies, also in psoriasis.

## 5. Conclusions

Gal-3 level may be a factor affecting the course of psoriasis and useful in prediction of cardiometabolic comorbidities, especially in patients with long history of the disease or obesity. Gal-3 probably cannot serve as an indicator of psoriasis severity, nor treatment effectiveness. Patients with low serum gal-3 and short history of psoriasis are presumably at greater risk of diabetes mellitus. In obese patients with long-lasting psoriasis gal-3 may exert beneficial influence against lipid aberrations or perhaps further cardiovascular disorders development.

## Figures and Tables

**Table 1:** Baseline characteristics of patients and control group.

Parameters	Control Group (*n* = 11)	Patients Group (*n* = 31)
Age [years]	51.4 ± 9.61	55.2 ± 15.6
Height [cm]	167 ± 9.3	172 ± 10.3
Weight [kg]	74.6 ± 21.3	85.2 ± 21.3
BMI [kg/m^2^]	25.6 ± 6.53	28.5 ± 6.53

**Table 2:** Basal characteristic of the patients’ group before and after total treatment.

Parameter	Before Treatment	After Treatment
PASI	17.15 ± 7.92	4.31 ± 2.06 ***
Hemoglobin [g/dL]	13.55 ± 1.71	13.21 ± 1.48
RBC [x10^3^/mL]	4.37 ± 0.57	4.29 ± 0.48
WBC [x10^3^/mL]	7.64 ± 1.88	6.63 ± 1.61 *
PLT [x10^3^/mL]	255 ± 64.5	231.7 ± 59.2
Glucose [mg/dL]	85 (53–215)	88.5 (55–140)
CRP [mg/L]	5.13 (1–34.7)	1.81 (0.5–15) **
ALT [U/L]	24.87 ± 9.05	21.03 ± 10.55
AST [U/L]	21.5 (14–86)	19 (12–52)
Total Chol [mg/dL]	171.6 ± 38.2	170.4 ± 35.6
TG [mg/dL]	137.1 ± 55.1	120.1 ± 46.62

Bold and */**/***—means the existence of statistically significant difference between values after and before treatment with *p* < 0.05; <0.01; <0.001, respectively.

## Data Availability

Data available upon the request.

## Supplementary Materials

The following are available online at https://www.mdpi.com/article/10.3390/biology11010088/s1, Table S1. Selected characteristic of the study subgroups depending on the drug. Table S2. Selected characteristic of the study subgroups depending on PASI. Table S3. Selected characteristic of the study subgroups depending on BMI. Table S4. Selected characteristic of the study subgroups depending on galectin level. Table S5. Characteristic of the study subgroups depending on the length of psoriasis history. Table S6. Selected correlations before and after treatment with reference to duration of psoriasis (**a**) and the galectin-3 concentration threshold (**b**). Figure S1. Correlations between gal-3 level and selected parameters before treatment with use of Spearman’s rank correlation. Figure S2. Correlations between selected parameters and serum galectin-3 level in patients before and after treatment with acitretin (ACY) and methotrexate (MTX) with use of Spearman’s rank correlation. Figure S3. Correlations between gal-3 level and selected parameters after treatment with use of Spearman’s rank correlation. Figure S4. Gal-3 concentrations in controls and patients before and after the treatment with methotrexate (**a**) and acitretin (**b**).
