# Supplementary material for: Galectin-3 as a Novel Multifaceted and Not Only Cardiovascular Biomarker in Patients with Psoriasis with Regard to Systemic Treatment—Preliminary Data
Source: Biology (Basel). 2022 Jan 7;11(1):88. doi: 10.3390/biology11010088 (PMC8773359; doi:10.3390/biology11010088)
Supplement: Supplementary file 1 [file biology-11-00088-s001.zip › biology-1502967-supplementary.pdf]

Table S1. Selected characteristic of the study subgroups depending on the drug.
Parameters with "1" mean the value before treatment and with "2" – after treatment.

**Parameter**	**Acitretin (*n* = 13)**	**Methotrexate (*n* = 18)**
PASI 1	**20.3±7.54**	**14.6±7.05 ***
PASI 2	**6.42±3.65 ^^^**	**3.14±1.54 ** ^^^**
Years	19 (2 – 58)	11 (1-63)
Current time (months)	2 (1 – 24)	3 (1 – 24)
Age	50.7±18.5	57.6±14.1
Body Weight [kg]	85.1±19.5	86.1±23.2
Height [cm]	**178±5.74**	**167.1±11.2****
BMI	27±6.01	30.4±6.69
Cigarette smoking	8/5	7/11
HGB 1	13.3±1.86	13.7±1.22
HGB 2	13.3±1.99	13.1±1.18
RBC 1	4.31±0.71	4.41±0.32
RBC 2	4.42±0.68	**4.15±0.31 ^**
WBC 1	7.09±1.11	8.07±2.11
WBC 2	6.56±1.25	**6.67±1.74 ^**
PLT 1	249±91.8	260±57.5
PLT 2	221±58.3	240±58.6
GLU 1	78 (53 – 125)	*92 (53 – 215) **
GLU 2	87 (55 – 110)	90 (65 – 140)
**CRP 1**	**6.35 (1 – 29.6)**	**3.55 (1 – 34.7)**
**CRP 2**	**1.87 (1 – 15) ^**	**1.94 (0.5 – 5.8) ^**
ALT 1	19 (10 – 48)	27 (12 – 46)
ALT 2	16 (9 – 42)	23 (12 – 49)
AST 1	21 (14 – 86)	21 (15 – 47)
AST 2	16 (12 – 39)	20 (13 – 52)
CHOL 1	154±36.8	**185±35.1 ***
CHOL 2	174±47.2	168±30.9
TG 1	122±51.1	143±62.8
TG 2	112±48.5	126±46.1

*/**/*** - means the existence of statistically significant difference between ACY vs MTX subgroup

^/^^/^^^ - means statistical differences in each parameter before and after treatment

Table S2. Selected characteristic of the study subgroups depending on PASI.

Parameters with "1" mean the value before treatment and with "2" – after treatment.

Parameter	PASI I (*n* = 8)	PASI II (*n* = 12)	PASI III (*n* = 11)
Family history	3/5	6/6	7/4
Sun's impact	6/2	6/6	4/7
PASI 1	**8.75±1.92**	**14.3±2.67 *****	**26.1±4.24 ***** ###
PASI 2	**2.95±1.9 ^^^**	**4.01±2.88 * ^^^**	**6.48±3.67 *** # **^^^**
Psoriasis history [yrs]	24.5 (2 – 52)	**8 (1 – 63) ***	24 (6 – 58)
Current time [months]	2.5 (1 – 10)	3 (1 – 24)	2.1 (1 – 20)
City/village	3/5	2/10	6/5
Age [yrs]	55.9±5.33	51.1±21.1	56.2±15.9
Body Weight [kg]	**87.1±19.8**	**47.8±12 ***	**95.9±25 *** ##
Height [cm]	170±10.3	170±11.9	175±7.58
BMI	**30.1±6.1**	**25.9±4.14 ***	**31.3±7.86** #
Cigarette smoking	4/4	6/6	4/7
HGB 1	14.4±1.34	13.8±1.53	12.95±1.92
HGB 2	13.5±0.92	13.4±1.99	12.8±1.08
RBC 1	4.61±0.48	4.35±0.63	4.29±0.54
RBC 2	4.33±0.32	4.28±0.65	4.31±0.38
WBC 1	7.62±2.24	7.55±1.91	7.68±1.7
WBC 2	6.64±1.65	6.48±1.38	7.05±1.83
PLT 1	230±41.6	259±71.4	260±98.8
PLT 2	226±59.7	241±65.7	224±62.1
GLU 1	94.5 (78 – 135)	83.5 (69 – 215)	84.3 (53 – 129)
GLU 2	88.5 (81 – 125)	88 (65 – 120)	87 (55 – 142)
CRP 1	**2.08 (1 – 7.3)**	**4.04 (1 – 7.2)**	**16.1 (6.22 – 34.7) ***** ##
CRP 2	**1.45 (1 – 2.46)**	**1.59 (0.5 – 13.9) ^**	**4.42 (1.2 – 15) *** # **^^^**
ALT 1	26±13.3	18.8±5.96	29.8±13.9
ALT 2	20.3±6.92	19.6±4.74	23.2±14.1
AST 1	21.1±4.36	21.1±4.8	31.3±20.6
AST 2	20.1±5.91	20±4.55	24.8±13.3
CHOL 1	177±43.4	175±47.8	161±28.9
CHOL 2	159±35.6	171±53.4	174±23.2
TG 1	168±73.5	138±71.4	116±40.9
TG 2	135±54.3	124±71.1	106±35.9

*** - means the existence of statistically significant difference between PASI I vs PASI II / III

- statistical differences between PASI II vs PASI III

^/^^/^^^ - means statistical differences in each parameter before and after treatment

Table S3. Selected characteristic of the study subgroups depending on BMI.

Parameters with "1" mean the value before treatment and with "2" – after treatment.

Parameter	BMI I (*n* = 10)	BMI II (*n* = 10)	BMI III (*n* = 11)
PASI 1	15.1±6.53	17.3±7.83	18±7.55
PASI 2	2.5 (1.8 – 15)	4.6 (1.8 – 8)	3.6 (0.7 – 8.1)
Psoriasis history [yrs]	**7 (2 – 63)**	**20.5 (5 – 30)** *	**33 (1 – 58)** *
Current time (months)	3 (1 – 24)	3 (1 – 24)	2 (1 – 20)
Age [yrs]	48.4±19.7	54.7±20.5	56.7±6.12
Body Weight [kg]	**63.9±5.24**	**81.4±9.99** ***	**105±17.1** *** ###
Height [cm]	171±10.2	173±10.9	171±9.75
BMI	**22.2±2.23**	**27.2±1.87** ***	**36±3.74** *** ###
Cigarette smoking	5/5	**7/4**	**3/9** #
HGB 1	13.7±1.53	13.2±1.87	13.8±1.62
HGB 2	13.1±1.7	13.2±1.73	13.2±1.01
RBC 1	4.3±0.49	4.29±0.64	4.52±0.56
RBC 2	4.17±0.43	4.34±0.6	4.33±0.39
WBC 1	7.18±2.12	6.87±1.4	8.33±1.87
WBC 2	6.1±1.42	6.6±1.32	7.08±1.94
PLT 1	249±83.8	257±76.5	247±66.8
PLT 2	222±75.9	246±47.8	225±56.5
GLU 1	80 (53 – 215)	81 (69 – 97)	**103 (53 – 135)** *
GLU 2	86 (55 – 112)	88.5 (78 – 95)	101 (67 – 140)
CRP 1	3.67 (1 – 16.1)	4.14 (1 – 28.1)	7.30 (1.48 – 34.7)
CRP 2	**1.2 (0.5 – 2.4)**	2.59 (1 – 13.6)	**2.48 (1 – 6) ^^**
ALT 1	19.9±9.87	18.2±7.11	**33.5±11.1** **
ALT 2	17.1±4.07	16.1±6.19	**28.3±11.2** **
AST 1	20 (15 – 86)	18 (14 – 29)	25 (15 – 47)
AST 2	18.5 (14 – 39)	16 (13 – 27)	22 (13 – 52)
CHOL 1	158±47.5	171.2±31.4	182±38.9
CHOL 2	156±43.5	186±38.9	167±32.6
TG 1	110±76.8	131±45.7	**167±55.2** *
TG 2	101±64.7	126±56.8	**131±38.6 ^**

*** - means the existence of statistically significant difference between BMI I vs BMI II / III

- statistical differences? between BMI II vs BMI III

^/^^/^^^ - mean statistical differences in each parameter before and after treatment

Table S4. Selected characteristic of the study subgroups depending on galectin level.

Parameters with "1" mean the value before treatment and with "2" – after treatment.

Parameter	Galectin Low	Galectin High
PASI 1	17.69±9.11	16.5±6.58
PASI 2	5.07±2.88	3.55±1.95
Psoriasis history [yrs]	23 (2 – 40)	10 (1.8 – 63)
Age [yrs]	51.53±16.04	58.8±14.7
Body Weight [kg]	88.37±18.99	82.87±23.69
Height [cm]	174.5±7.24	169.6±12.45
BMI	29±5.69	28.89±7.47
Cigarette smoking	8/7	7/8
HGB 1	13.6±1.98	13.5±1.46
HGB 2	13.47±1.51	12.95±1.47
RBC 1	4.47±0.61	4.26±0.53
RBC 2	4.47±0.47	4.12±0.44
WBC 1	7.56±1.63	7.74±2.16
WBC 2	7.09±1.39	6.17±1.72
PLT 1	263.1±66.52	246.9±83.41
PLT 2	237.7±54.3	225.8±70.98
GLU 1	80 (69 – 135)	86 (53 - 215)
GLU 2	91 (78 – 125)	86 (55 – 215)
CRP 1	4 (1 – 29.6)	7 (1 – 34.7)
CRP 2	1.87 (1 – 15)	1.94 (0.5 – 6)
ALT 1	24.13±12.4	25.6±11.93
ALT 2	19.53±10.12	22.53±9.05
AST 1	23 (14 - 34)	20 (17 – 86)
AST 2	17 (12 – 29)	19 (13 – 52)
CHOL 1	175.6±34.5	167.7±46.14
CHOL 2	183.4±36.11	157.4±39.12
TG 1	**157.5±59.83**	**116.7±65.63 ***
TG 2	**135.8±48.26**	**104.3±32.12 ***

* - means the existence of statistically significant difference

Table. S5. Characteristic of the study subgroups depending on the length of psoriasis history.

Parameters with "1" mean the value before treatment and with "2" – after treatment.

Parameter	Short history: <20 yrs (*n* = 15)	Long history: >20 yrs (*n* = 16)
PASI I	16.07±6.55	18.13±6.87
PASI II	2.6 (1.8 – 15)	3.6 (0.7 – 8.4)
Years	**6 (1 – 20)**	**33 (21 – 63)** ***
Age [years]	52.07±18.73	58.27±11.42
Body Weight [kg]	81.17±21.2	90.07±20.51
Height [cm]	172.2±10.19	171.9±10.78
BMI	27.39±6.62	30.49±6.27
Cigarette smoking	8/7	7/9
HGB 1	13.74±2	13.36±1.41
HGB 2	13.38±1.94	13.04±0.86
RBC 1	4.4±0.7	4.33±0.43
RBC 2	4.32±0.63	4.26±0.29
WBC 1	7.68±1.77	7.62±2.06
WBC 2	6.93±1.44	6.87±1.77
PLT 1	*274.8±55.15*	*235.2±70.22*
PLT 2	228.3±60.4	235.1±45.51
GLU 1	83 (69 – 215)	92 (53 – 135)
GLU 2	87 (78 – 140)	91 (55 – 125)
CRP 1	5.14 (1 – 34.7)	5.13 (1 – 29.6)
CRP 2	1.87 (0.5 – 15)	2.37 (1 – 6)
ALT 1	22.93±11.11	26.8±12.83
ALT 2	19.87±7.77	22.2±10.22
AST 1	22.93±8.74	26.47±12.95
AST 2	21.33±8.82	22±8.67
CHOL 1	172.8±43.32	170.5±38.38
CHOL 2	168.7±37.27	172.1±38.98
TG 1	134.6±57.86	139.7±54.61
TG 2	118.3±56.38	121.8±37.21

***- means the existence of statistically significant difference (p<0.001)

Table S6. Selected correlations before and after treatment with reference to duration of psoriasis (a) and the galectin-3 concentration threshold (b)

a)

	**Short Length <20 yrs (*n* =1 5)**		**Long Length >20 yrs (*n* = 16)**		
	Disease Length	Glucose	Disease Length	Chol Total	TG
**Galectin Before**	-0.576/*	0.619/**	0.608/*	-0.618/**	-0.725/**
**Galectin After**	-0.571/*	0.195/NS	0.46/0.062	-0.626/**	-0.385/0.11

b)

	**Galectin Low**			**Galectin High**		
	Body weight	BMI	Glucose	Body weight	BMI	Glucose
**Galectin Before**	**0.488/***	**0.662/****	**0.693/****	-0.067/NS	0.041/NS	0.082/NS
**Galectin After**	0.130/NS	0.246/NS	0.124/NS	0.242/NS	0.211/NS	-0.109/NS

*/**- means the existence of statistically significant difference between the parameters with p <0.05; < 0.01, respectively

NS- non-signifant

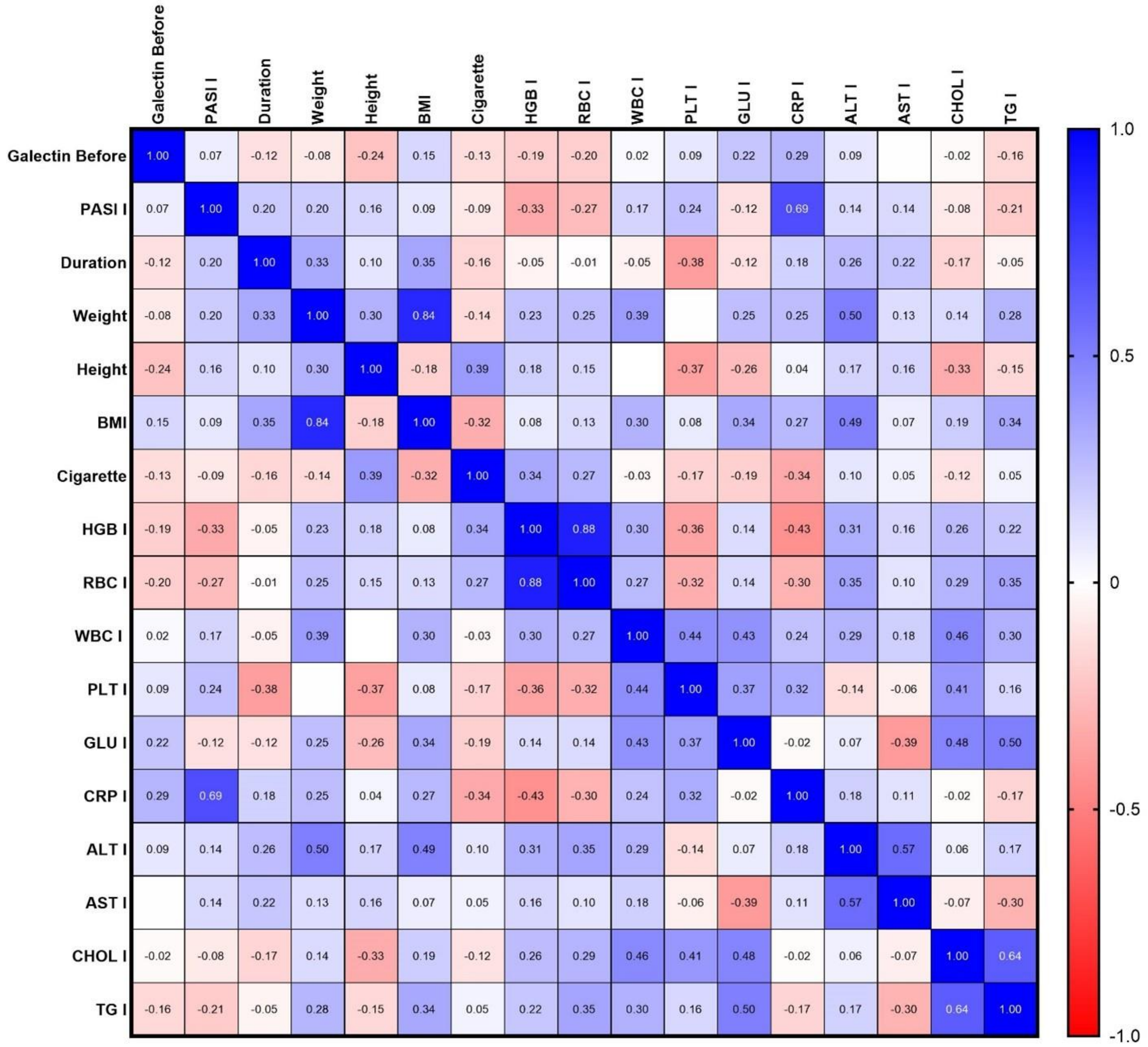

Figure S1. Correlations between gal-3 level and selected parameters before treatment with use of Spearman's rank correlation.

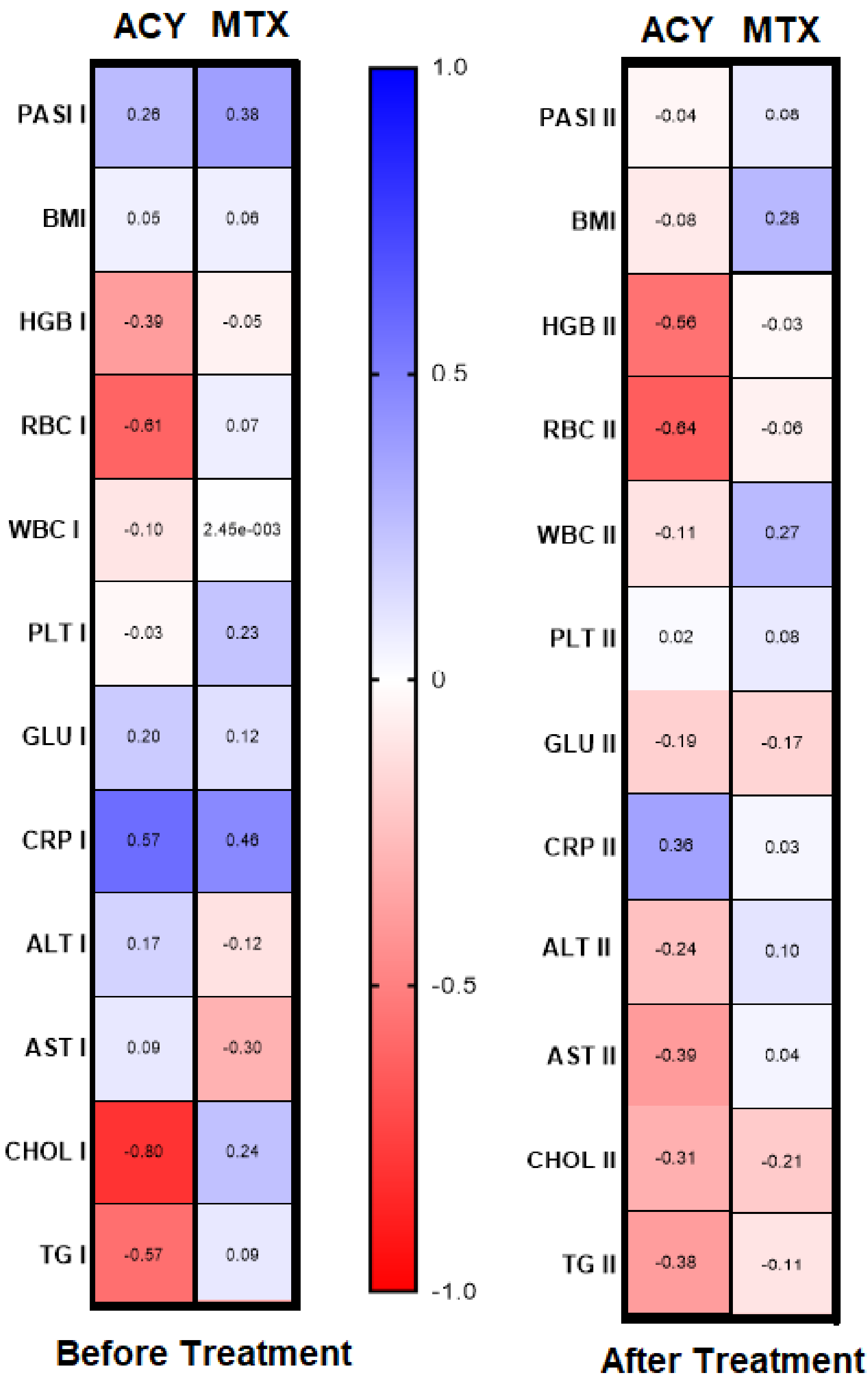

Figure S2. Correlations between selected parameters and serum galectin-3 level in patients before and after treatment with acitretin (ACY) and methotrexate (MTX) with use of Spearman's rank correlation.

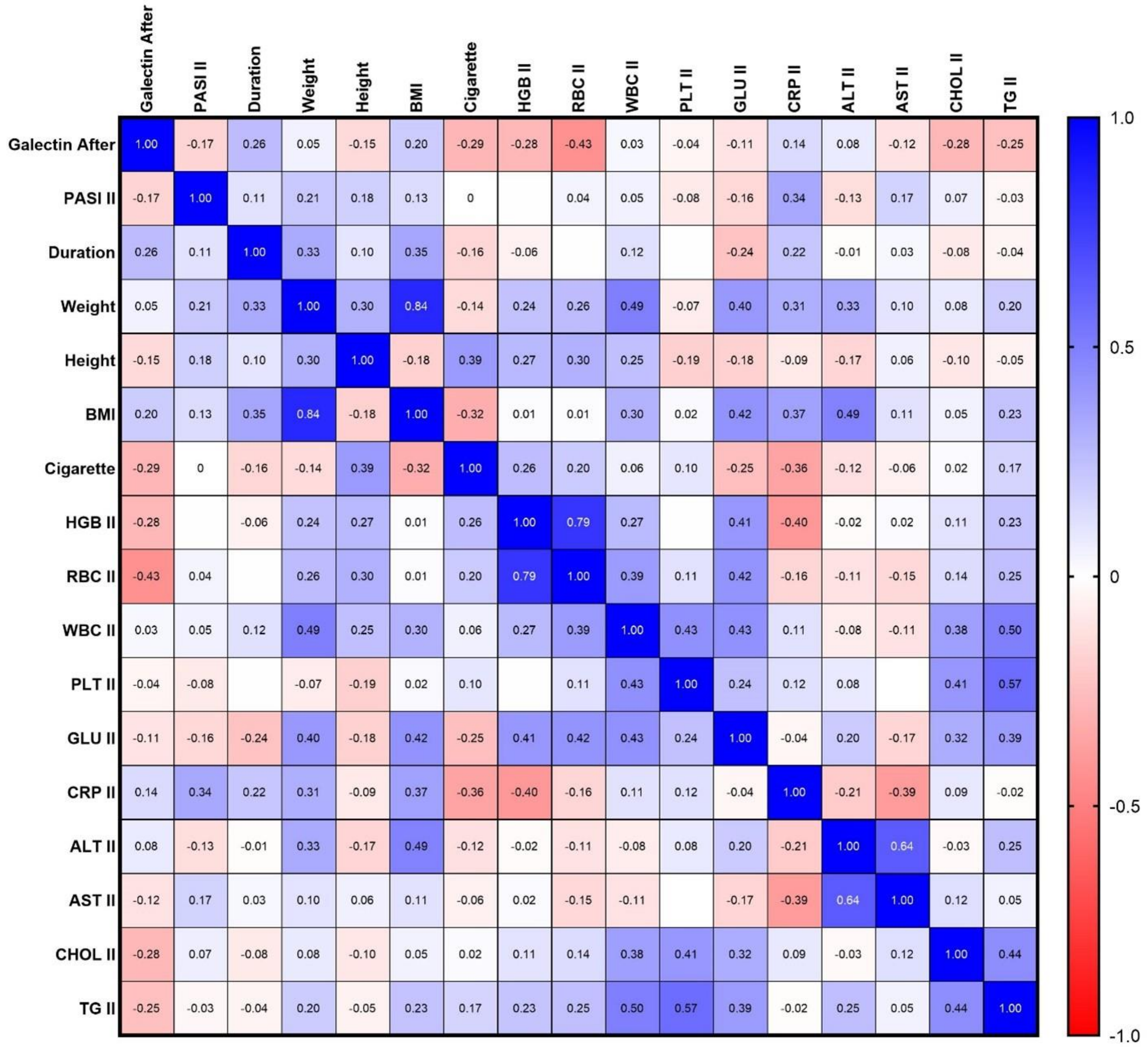

Figure S3. Correlations between gal-3 level and selected parameters after treatment with use of Spearman's rank correlation.

(a) (b)

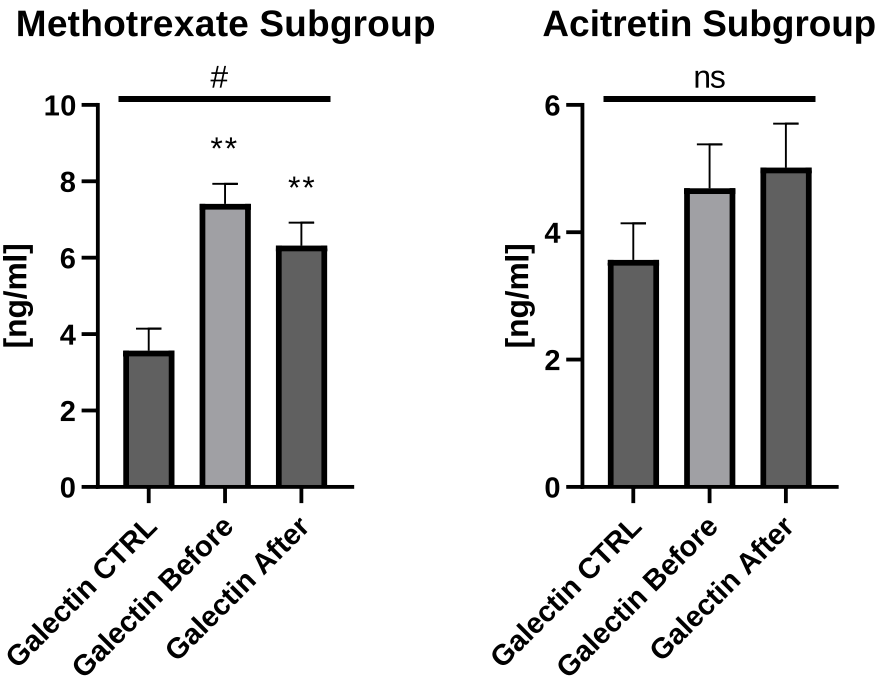

Figure S4. Gal-3 concentrations in controls and patients before and after the treatment with methotrexate (a) and acitretin (b). *// - means the existence of statistically significant difference between patients single group compared to control group with p <0.05; < 0.01, respectively. # - shows the statistical significance between control group and marked patients' subgroups when compared using ANOVA with p <0.05.**

NS, non-significant
